# Beyond Integrin Activation: Kindlin-3 as an Organiser of Immune Receptor Signalling

**DOI:** 10.3390/ijms27198770

**Published:** 2026-09-30

**Authors:** Zheng-Kai Teng, Suet-Mien Tan

**Affiliations:** School of Biological Sciences, Nanyang Technological University, 60 Nanyang Drive, Singapore 637551, Singapore; zhengkai001@e.ntu.edu.sg

**Keywords:** kindlins, cell adhesion, cytokine signalling

## Abstract

Kindlins are best known for their roles in integrin regulation, but studies across the family have revealed additional functions in receptor trafficking, stability and signalling. In this review, we examine these non-canonical functions with a focus on kindlin-3 and its emerging role in cytokine receptor regulation. Kindlin-3 is the predominant kindlin in haematopoietic cells and is essential for integrin-dependent leukocyte adhesion and platelet function. Beyond these established roles, kindlin-3 participates in bidirectional integrin regulation, promotes integrin clustering and associates with signalling adaptors that couple adhesion to downstream cellular responses. A recent study further reported that kindlin-3 binds interleukin-2 receptor β (IL-2Rβ) through its F0 subdomain and promotes IL-2Rβ retention at the plasma membrane in a manner dependent on the integrin lymphocyte function-associated antigen 1 (LFA-1). Loss of kindlin-3 accelerates IL-2Rβ internalisation; reduces the persistence of interleukin-2 (IL-2)-induced signal transducer and activator of transcription 5 (STAT5), AKT and extracellular signal-regulated kinase 1/2 (ERK1/2) phosphorylation; and increases natural killer cell susceptibility to apoptosis under limiting IL-2 conditions, suggesting a possible role of kindlin-3 in sustaining cytokine responsiveness in immune cells. We further consider structural features and post-translational mechanisms that may influence receptor organisation, including the F3 integrin-binding subdomain, F1 loop, pleckstrin homology domain and phosphorylation of kindlin-3. Finally, we discuss whether related receptor-organising functions extend to interleukin-15 (IL-15) signalling, other kindlin paralogues and distinct tissue contexts. Together, the available evidence broadens the established role of kindlin-3 in immune cells and raises important questions about how kindlins coordinate integrins with other signalling receptors.

## 1. Introduction: From Integrin Activation to Receptor Organisation

Cellular responses to the extracellular environment depend on how receptors, adaptor proteins and downstream signalling components are organised at the plasma membrane. Integrins are central to this process, functioning not only as adhesion receptors but also as bidirectional signalling platforms that regulate cell migration, survival, proliferation and immune-cell activation [1,2]. The classical view of kindlins places them within this integrin-regulatory framework. Kindlins act alongside talin at integrin β cytoplasmic tails and make complementary contributions to integrin conformational regulation, ligand-dependent adhesion and downstream signalling [3,4,5].

Although best characterised as integrin co-regulators, kindlins are increasingly recognised to have broader, including integrin-independent, roles in cellular signalling [5]. Studies across the kindlin family have shown that kindlins can associate with non-integrin receptors, regulate receptor stability and trafficking and participate in multiprotein signalling complexes [6,7,8]. These findings suggest that kindlins should not be considered solely as regulators of integrin activity, but also as context-dependent organisers of receptor signalling and crosstalk.

Kindlin-3 provides a particularly relevant haematopoietic example of this broader principle. As the predominant kindlin family member in leukocytes and platelets, kindlin-3 is best known for regulating β1, β2 and β3 integrins, with essential roles in leukocyte adhesion, immune-cell trafficking and platelet function [3,9,10,11,12]. Emerging evidence suggests that kindlin-3 may also contribute to the coordination of integrins with non-integrin receptors. A recent study showed that kindlin-3 binds the interleukin-2 receptor β subunit (IL-2Rβ) through its F0 subdomain and promotes integrin lymphocyte function-associated antigen 1 (LFA-1)-dependent retention of IL-2Rβ at the plasma membrane, thereby prolonging interleukin-2 (IL-2) signalling and supporting natural killer cell survival under limiting cytokine conditions [13], suggesting a wider role of kindlin-3 in immune cell function.

In this review, we place kindlin-3 within this expanded conceptual framework. We first consider non-canonical signalling and receptor-regulatory functions across the kindlin family, including evidence for functional overlap and isoform specificity. We then examine kindlin-3 in the context of bidirectional haematopoietic integrin regulation, receptor clustering and integrin-associated signalling complexes. Particular attention is given to the emerging relationship between integrin LFA-1, kindlin-3 and IL-2Rβ, together with the structural and post-translational mechanisms that may regulate receptor organisation and cytokine responsiveness. Finally, we consider whether related receptor-organising functions extend across different kindlin paralogues, receptor systems and tissue contexts, and highlight outstanding mechanistic and translational questions. This review was supported by a structured literature search, described in Section 8.

## 2. Kindlin Family Overview: Signalling Roles Beyond Canonical Integrin Activation

The kindlin family comprises kindlin-1, kindlin-2 and kindlin-3, encoded by *FERMT1*, *FERMT2* and *FERMT3*, respectively. In addition to integrins, kindlins interact with a diverse range of proteins, including growth factor receptors, intracellular kinases, transcriptional regulators and trafficking proteins (Table 1). Some of these interactions remain closely linked to integrin-dependent adhesion and signalling, whereas others regulate receptor stability, trafficking or intracellular signalling independently of a demonstrated role in integrin regulation.

To understand these diverse signalling functions in a structural context, it is useful to consider the distinctive organisation of the kindlin FERM domain. Canonical FERM domains, exemplified by protein 4.1 and ezrin, radixin and moesin (ERM) proteins, comprise F1, F2 and F3 subdomains arranged in a compact cloverleaf-like structure [36]. Kindlins retain this core organisation but, like talin, possess an additional N-terminal F0 subdomain and an extended insertion within F1 [36,37]. A distinctive feature of kindlins is the insertion of a pleckstrin homology (PH) domain within F2, which provides an additional phosphoinositide-binding interface [36,38]. Beyond the FERM domain, protein 4.1 contains additional FERM-adjacent, spectrin–actin-binding and C-terminal regions [39]. ERM proteins contain an α-helical region followed by a C-terminal F-actin-binding domain [40], whereas talin couples its FERM head to a large C-terminal rod composed of multiple helical bundles [36]. Kindlins consist predominantly of an F0–F3 architecture, including a polylysine-containing insertion within F1 and a PH domain inserted within and bisecting the F2 subdomain [38,41,42].

### 2.1. Kindlin-1 in Epithelial Receptor Signalling

Kindlin-1 is predominantly expressed in epithelial tissues, where it contributes to cell adhesion and migration, as well as the preservation of tissue integrity [5]. Beyond these integrin-associated functions, evidence indicates that kindlin-1 can regulate non-integrin receptor signalling and other intracellular signalling processes.

Epidermal growth factor receptor (EGFR) provides a well-characterised example. Kindlin-1 associates with EGFR following epidermal growth factor (EGF) stimulation through an interaction that does not require integrin engagement [7]. Loss of kindlin-1 reduces EGFR abundance, downstream EGF signalling and EGF-induced keratinocyte migration. Mechanistically, kindlin-1 limits the lysosomal degradation of EGFR, thereby maintaining a pool of signalling-competent receptors [7]. Kindlin-1 can therefore regulate receptor stability independently of its interaction with integrin cytoplasmic tails.

A second example is provided by transforming growth factor β (TGF-β) signalling. In colorectal cancer cells, kindlin-1 has been detected in a complex containing Smad anchor for receptor activation (SARA), TGF-β receptor I (TβRI) and Smad3 [15]. Formation of this complex promotes Smad3 activation and downstream TGF-β signalling [15]. Beyond membrane-associated signalling, kindlin-1 also interacts with polo-like kinase 1 at centrosomes during the G2/M phase, supporting mitotic spindle formation and cell survival [14]. These findings indicate that kindlin-1 can regulate both receptor-associated and intracellular signalling complexes.

### 2.2. Kindlin-2 as an Organiser of Receptor Signalling

Kindlin-2 is ubiquitously expressed [5] and has the most extensively characterised repertoire of non-integrin protein partners among the three kindlins. Rather than discussing each partner individually, its non-canonical functions can be broadly grouped into two closely related activities: regulation of receptor abundance and organisation of receptor crosstalk.

Kindlin-2 also influences EGFR availability and signalling. In breast cancer cells, its association with EGFR reduces receptor ubiquitination and degradation, preserving EGFR abundance and supporting EGF-driven migration [6]. Kindlin-2 can also regulate EGFR at the transcriptional level. In glioma cells, kindlin-2 associates with β-catenin and Y-box-binding protein 1 (YB-1) to enhance EGFR transcription and promote tumour progression [25]. Together, these studies demonstrate that kindlin-2 can regulate the same receptor at multiple levels, including gene expression, protein stability and downstream biological responses.

Kindlin-2 also has important functions in vascular endothelial cells that extend beyond classical integrin activation. In endothelial cells, kindlin-2 regulates the surface abundance of the ectonucleotidases CD39 and CD73 through a clathrin-dependent trafficking mechanism, thereby influencing extracellular nucleotide metabolism and haemostatic responses [8]. Kindlin-2 also contributes to endothelial barrier integrity by localising to adherens junctions, where it associates with β- or γ-catenin and cortical actin to stabilise cell–cell contacts [43]. A more recent study has linked endothelial kindlin-2 to Notch1 signalling and physiological angiogenesis [44], while flow-dependent phase separation of kindlin-2 has been implicated in the maintenance of endothelial integrity and vascular stability [45]. Together, these findings illustrate that kindlin-2 can regulate membrane trafficking, junctional organisation and receptor signalling in the vascular endothelium, further extending its functions beyond direct control of integrin activation.

Kindlin-2 has also been implicated in broader intracellular signalling processes, including transcriptional regulation through β-catenin and T-cell factor 4 (TCF4) and regulation of the Hippo pathway through MOB kinase activator 1 (MOB1) degradation [24,27]. Although mechanistically distinct, these functions reinforce the view of kindlin-2 as a multifunctional signalling adaptor and suggest that the regulation of receptor organisation, trafficking and turnover represents an important non-canonical dimension of kindlin biology.

Evidence from renal tubular epithelial cells further implicates kindlin-2 in TGF-β signalling. The association of kindlin-2 with TβRI and Smad3 promotes Smad activation and fibrotic responses [22], indicating that kindlin-2 can associate with both a non-integrin receptor and its downstream signalling machinery.

More recent work on triple-negative breast cancer provides a particularly direct example of receptor coordination by kindlin-2. By engaging both β1 integrin and TβRI, kindlin-2 stabilises a ternary β1 integrin–kindlin-2–TβRI complex [23]. Disruption of kindlin-2 destabilises both receptors, weakens downstream signalling and suppresses tumour progression and metastasis [23]. This provides direct evidence that a kindlin can organise a membrane-proximal complex containing both an integrin and a structurally distinct signalling receptor.

The β1 integrin–kindlin-2–TβRI complex is particularly relevant to the receptor-organising model developed in this review. It provides a precedent for kindlin-dependent coordination of receptor proximity, stability and signalling competence, rather than regulation solely through changes in integrin affinity. Kindlin-2 may therefore act as a molecular scaffold that couples integrin engagement to growth factor receptor signalling. This precedent provides a useful conceptual basis for examining whether kindlin-3 similarly coordinates integrin LFA-1 with IL-2Rβ in haematopoietic cells.

### 2.3. Kindlin-3 and Haematopoietic Signalling Complexes

**Figure 1:**
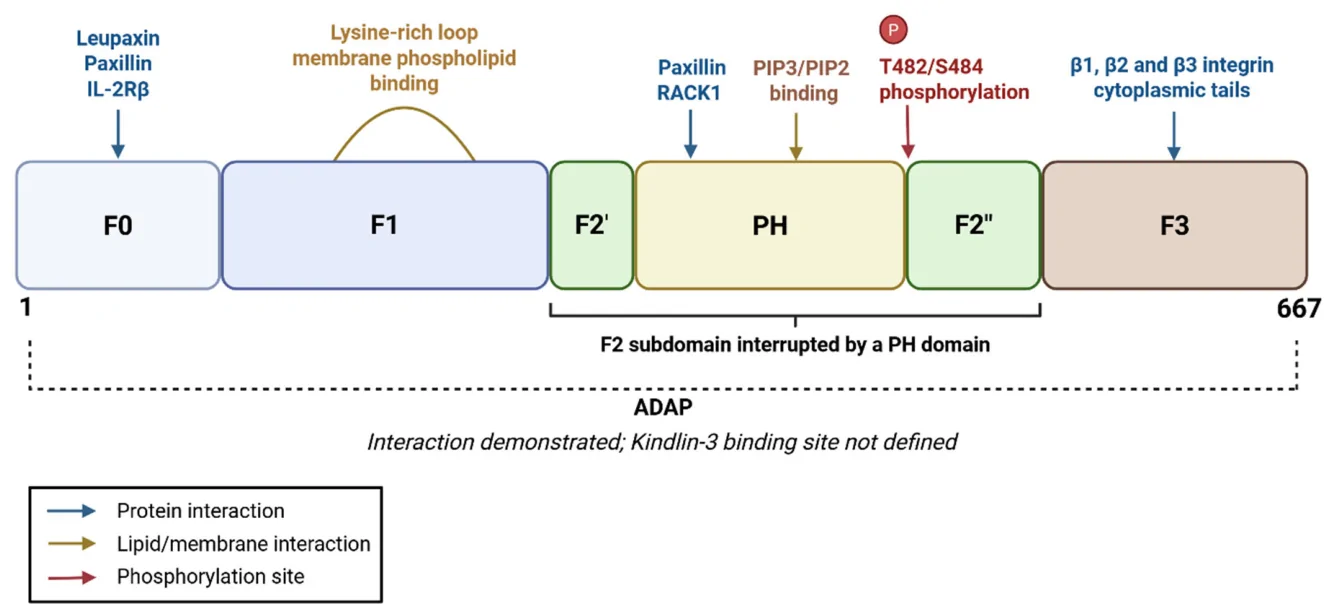
Domain architecture and selected interaction and regulatory sites of kindlin-3. Kindlin-3 comprises an N-terminal F0 subdomain followed by the F1, F2 and F3 subdomains, with a pleckstrin homology (PH) domain inserted within F2. Experimentally mapped protein interactions include leupaxin, paxillin and IL-2Rβ at the F0 subdomain; paxillin and RACK1 at the PH-containing region; and β1, β2 and β3 integrin cytoplasmic tails at the F3 domain. The lysine-rich F1 loop and PH domain contribute to membrane association, with the PH domain binding phosphoinositides including PIP3 and PIP2. Thr482 and Ser484 are reported phosphorylation sites implicated in the regulation of kindlin-3 function. ADAP associates with kindlin-3, although the corresponding kindlin-3 interaction site has not been defined. Only selected interactions relevant to the present review are shown. Abbreviations: IL-2Rβ, interleukin-2 receptor β; RACK1, receptor for activated C kinase 1; PIP3, phosphatidylinositol 3,4,5-trisphosphate; PIP2, phosphatidylinositol 4,5-bisphosphate; ADAP, adhesion and degranulation-promoting adaptor protein. Created in BioRender. Teng, Z.-K. (2026) https://BioRender.com/pi84c1b (accessed on 6 September 2026).

Kindlin-3 is expressed predominantly in haematopoietic cells, including leukocytes and platelets, although expression in endothelial cells has also been reported [46]. Compared with kindlin-1 and kindlin-2, relatively few non-integrin protein partners of kindlin-3 have been characterised. Nevertheless, kindlin-3 has been identified within multiprotein signalling assemblies in haematopoietic cells. Examples include receptor for activated C kinase 1 (RACK1) during β2 integrin outside-in signalling [32], adhesion and degranulation-promoting adaptor protein (ADAP) together with talin during platelet integrin αIIbβ3 activation [33], and the paxillin-family proteins leupaxin and paxillin in podosome organisation and platelet integrin signalling, respectively [34,35]. The modular architecture of kindlin-3 and selected experimentally mapped interaction and regulatory sites relevant to these functions are summarised in Figure 1.

**Figure 2:**
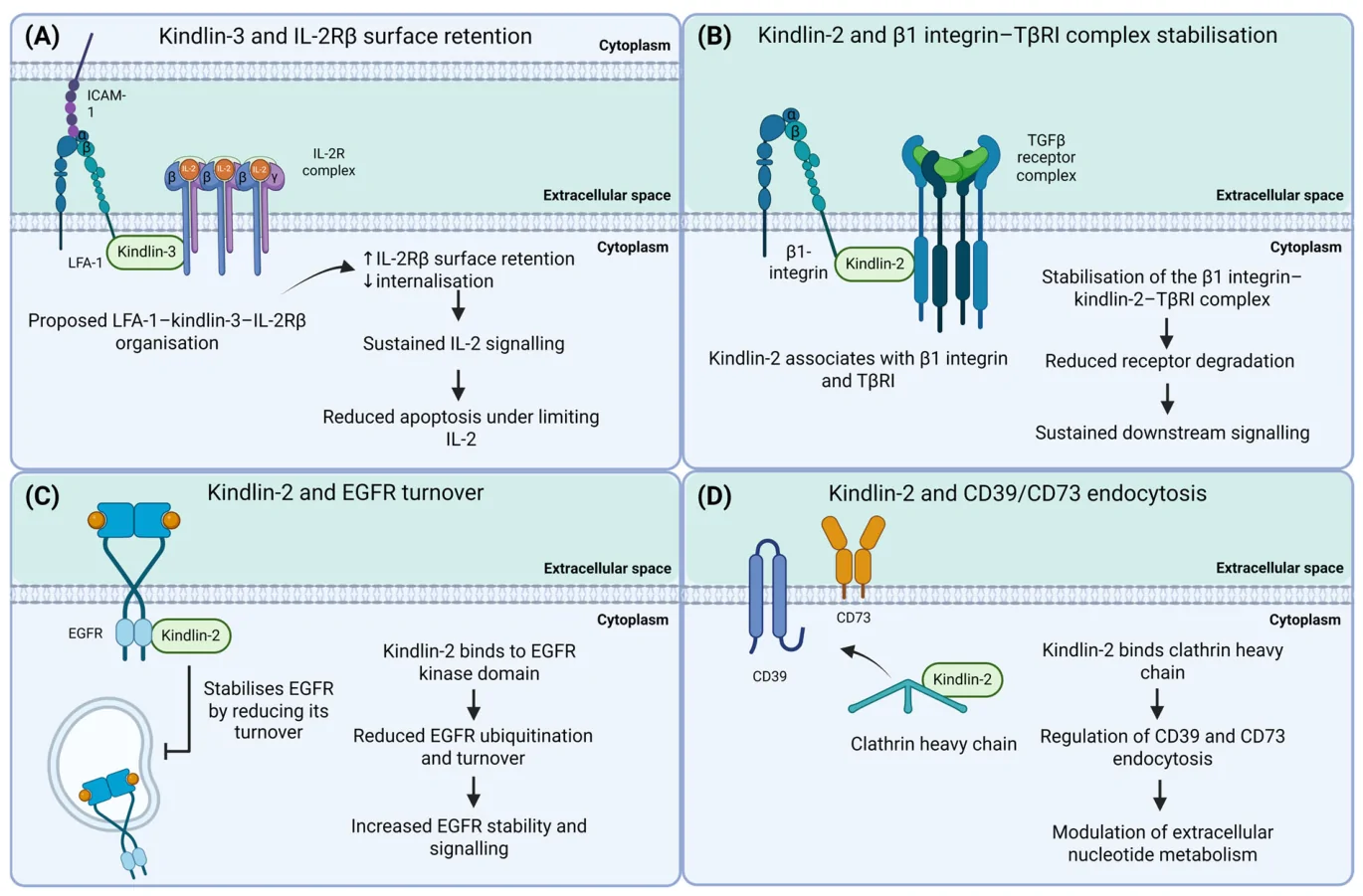
Kindlins as regulators of membrane protein organisation, trafficking and turnover. (**A**) Kindlin-3 associates with integrin LFA-1 and IL-2Rβ and promotes LFA-1-dependent retention of IL-2Rβ at the plasma membrane, reducing receptor internalisation and supporting sustained IL-2 signalling and cell survival under limiting IL-2 conditions. (**B**) Kindlin-2 associates with β1 integrin and TGF-β receptor I (TβRI), stabilising a β1 integrin–kindlin-2–TβRI complex and limiting receptor degradation, thereby supporting downstream TGF-β signalling. (**C**) Kindlin-2 interacts with epidermal growth factor receptor (EGFR) and reduces receptor ubiquitination and turnover, thereby maintaining EGFR abundance and EGF-dependent signalling. (**D**) Kindlin-2 associates with clathrin heavy chain and regulates CD39 and CD73 endocytosis, thereby influencing their cell-surface abundance and extracellular nucleotide metabolism. Abbreviations: LFA-1, lymphocyte function-associated antigen 1; IL-2Rβ, interleukin-2 receptor β; IL-2, interleukin-2; TGF-β, transforming growth factor β; EGF, epidermal growth factor. Created in BioRender. Teng, Z.-K. (2026) https://BioRender.com/2lc7n0k (accessed on 6 September 2026).

These interactions remain closely linked to integrin regulation but demonstrate that kindlin-3 functions within larger signalling assemblies rather than solely as an isolated β-integrin-binding protein. Direct regulation of non-integrin receptors remains comparatively underexplored. This gap provides the rationale for examining whether kindlin-3 can coordinate immune integrins with cytokine receptors and other membrane-associated proteins (Figure 2).

### 2.4. Functional Redundancy and Isoform Specificity Among Kindlins

The distinct phenotypes associated with loss of individual kindlin paralogues demonstrate substantial functional specialisation in vivo. Loss-of-function mutations in *FERMT1* cause Kindler syndrome, which is characterised by epithelial fragility affecting the skin and other epithelial tissues [47], whereas *FERMT3* mutations cause leukocyte adhesion deficiency type III (LAD-III), reflecting the essential role of kindlin-3 in haematopoietic integrin function [48]. In contrast, no equivalent human loss-of-function disorder has been described for *FERMT2*. In mice, complete loss of kindlin-2 causes peri-implantation lethality and severe defects in endoderm and epiblast adhesion [49]. Collectively, these observations indicate that the three kindlins are not functionally interchangeable at the organismal level, owing in part to their distinct tissue distributions and physiological roles.

Partial redundancy can nevertheless occur in cells or tissues in which multiple kindlin paralogues are co-expressed. In human keratinocytes, combined loss of kindlin-1 and kindlin-2 produces more severe defects in focal adhesion formation, spreading and adhesion than loss of either paralogue alone, indicating partial functional compensation [50]. Consistent with this, kindlin-2 expression can rescue epidermal adhesion defects caused by kindlin-1 deficiency in zebrafish [51]. Thus, functional redundancy among kindlins appears to be context-dependent and incomplete, with the extent of compensation determined by paralogue expression and cellular context.

## 3. Kindlin-3 in Haematopoietic Integrin Regulation and Signalling

Kindlin-3 is required for integrin function across multiple haematopoietic cell types. In leukocytes, it contributes to β1 and β2 integrin-dependent adhesion, spreading and recruitment to the vascular endothelium [10], whereas in platelets it is required for efficient integrin αIIbβ3 activation, fibrinogen binding, aggregation and haemostasis [11]. These functions extend beyond the initial establishment of active integrin states to include their reinforcement following ligand engagement [12]. Kindlin-3 can therefore be considered within a bidirectional regulatory framework in which intracellular adaptor engagement, extracellular ligand binding and cytoskeletal force cooperate to shape integrin conformation and signalling.

### 3.1. Bidirectional Regulation of Integrin Activation

Through its F3 subdomain, kindlin-3 binds the membrane-distal region of integrin β cytoplasmic tails. This site is distinct from the membrane-proximal region recognised by talin, allowing the two proteins to make complementary contributions to integrin regulation [52,53]. In leukocytes, talin-1 promotes integrin LFA-1 extension, whereas both talin-1 and kindlin-3 are required for the formation of the high-affinity conformation that supports firm adhesion [54]. In the classical inside-out model, intracellular signalling promotes the engagement of talin-1 and kindlin-3 with the integrin β cytoplasmic tail, thereby favouring conformational activation [2].

However, integrin activation is a bidirectional process in which extracellular ligand binding and tensile force transmission can influence receptor conformation and intracellular adaptor engagement. Ligand occupancy of platelet integrin αIIbβ3 has been shown to induce conformational changes associated with increased receptor activation [55]. In addition, single-molecule analysis of integrin α5β1 demonstrated that ligand binding can rapidly promote integrin extension and headpiece opening, whereas talin primarily stabilises the active conformation [56]. Mechanical force provides an additional layer of regulation, as actin-derived tension transmitted through β2 integrins can induce and stabilise active integrin LFA-1 conformations in a manner requiring intact talin- and kindlin-binding motifs [57]. Similar feedback regulation has been reported for integrin LFA-1. Intercellular adhesion molecule 1 (ICAM-1) binding induces the transient association of the membrane-proximal β2 cytoplasmic region with the N-terminal region of kindlin-3, disrupting the αL-β2 cytoplasmic clasp and promoting integrin unbending. These conformational changes favour talin-1 binding, while ligand-induced outside-in signalling enhances Rap1 activation, establishing a positive-feedback pathway that reinforces integrin LFA-1 activation [58]. In primary T cells under shear flow, ICAM-1 engagement similarly promotes rapid Rap1 activation and talin-1 recruitment, whereas higher Rap1 activation following chemokine stimulation promotes talin-1- and kindlin-3-dependent high-affinity integrin LFA-1 binding and firm arrest [59].

### 3.2. Integrin Clustering and Spatial Organisation

In addition to regulating integrin conformation, kindlin-3 contributes to the spatial organisation of β2 integrins at the cell surface. This is particularly evident in integrin clustering, which increases local receptor density and can support multivalent ligand binding, adhesion strengthening and signal propagation. Kindlin-3 was initially shown to promote microclustering of integrin LFA-1 following ICAM-1 engagement [32]. More recently, super-resolution imaging demonstrated that stimulation with interleukin-8 (IL-8) or N-formylmethionyl-leucyl-phenylalanine (fMLP) induces clustering of both integrin LFA-1 and macrophage-1 antigen (Mac-1) in suspended neutrophil-like cells [60]. This clustering was strongly impaired following kindlin-3 deletion but was largely preserved following talin-1 deletion, despite both proteins being required for full integrin activation [60]. These findings indicate that integrin affinity and clustering can be regulated as distinct processes, with kindlin-3 having a particularly important role in organising β2 integrins into nanoscale membrane clusters.

Membrane recruitment provides a plausible mechanism for this spatial function. The kindlin-3 PH domain interacts preferentially with phosphatidylinositol-(3,4,5)-trisphosphate and is necessary for efficient integrin LFA-1-dependent adhesion and lymphocyte migration [61]. During neutrophil arrest, kindlin-3 is recruited to the plasma membrane, where small clusters of high-affinity β2 integrins subsequently assemble within larger membrane regions enriched in kindlin-3 [62]. Deletion of the kindlin-3 PH domain also impairs integrin LFA-1 and Mac-1 clustering [60], further linking membrane recruitment of kindlin-3 with the spatial organisation of β2 integrins.

These observations indicate that kindlin-3 contributes to the spatial organisation of membrane receptors through mechanisms that are at least partly separable from its effects on integrin affinity. By coupling membrane localisation with β2 integrin clustering, kindlin-3 may help define membrane regions in which signalling proteins and other receptors are brought into proximity. These spatial functions may also provide a platform for the assembly of integrin-associated signalling complexes.

### 3.3. Integrin-Associated Signalling Complexes

Beyond its effects on integrin conformation and clustering, kindlin-3 associates with signalling and cytoskeletal proteins that couple integrin engagement to downstream cellular responses. RACK1 provides one example of this scaffold function. Kindlin-3 associates with RACK1 through its PH domain, while RACK1 can also bind the β2 integrin cytoplasmic tail, permitting formation of a ternary β2 integrin–kindlin-3–RACK1 complex [32]. In migrating T cells, kindlin-3 and RACK1 colocalise at the leading edge following integrin LFA-1 engagement of ICAM-1, consistent with a role for this complex in organising membrane-proximal signalling during cell adhesion and migration [32]. More recent work in neutrophils suggests that this complex is also mechanically regulated, with tensile force transmitted through integrin LFA-1 triggering kindlin-3-dependent redistribution of RACK1 and promoting local Orai1-dependent Ca^2+^ influx [63].

Kindlin-3 also contributes to β2 integrin outside-in signalling that controls cytoskeletal remodelling. In cells expressing integrin Mac-1, the reduction in kindlin-3 impaired ligand-induced phosphorylation of the spleen tyrosine kinase (Syk) and the guanine nucleotide exchange factor Vav1 even when integrin activation was bypassed experimentally [64]. This was accompanied by reduced activation of the Rho-family GTPases Rac1 and Cdc42 and defective cell spreading [64]. These data place kindlin-3 upstream of a Mac-1–Syk–Vav1–Rac1/Cdc42 signalling axis that links integrin engagement to actin reorganisation. However, direct physical interactions between kindlin-3 and Syk or Vav1 have not been demonstrated. Kindlin-3 may therefore support this pathway by organising integrins and associated signalling complexes rather than by directly binding each downstream component.

Kindlin-3-dependent organisation is further illustrated in podosomes, dynamic integrin-based adhesion structures found in myeloid cells. In osteoclasts, kindlin-3 deficiency disrupts actin organisation and impairs podosome maturation, organisation and subsequent assembly into podosome belts and sealing zones [65]. At the molecular level, kindlin-3 binds directly to the paxillin-family adaptor leupaxin through a loop in its F0 subdomain and promotes leupaxin recruitment to podosomes [34]. Leupaxin recruitment supports protein tyrosine phosphatase non-receptor type 12 (PTPN12)-dependent dephosphorylation of paxillin at Y31 and Y118, reducing podosome turnover and increasing podosome stability [34]. Notably, paxillin recruitment itself is largely independent of kindlin-3, indicating that kindlin-3 regulates paxillin signalling indirectly through leupaxin rather than simply controlling paxillin localisation [34]. Kindlin-3 can nevertheless interact directly with paxillin, and this interaction has also been implicated in supporting integrin αIIbβ3 activation in platelets [35].

Together, these studies demonstrate that the contribution of kindlin-3 to integrin signalling extends beyond the regulation of receptor conformation. Through interactions with scaffold and cytoskeletal adaptors, and through its contribution to downstream signalling pathways, kindlin-3 can couple ligand-engaged integrins to local signalling and actin-remodelling machinery. This provides a mechanism by which integrin engagement can be translated into the signalling and structural responses required for immune-cell adhesion, migration and function.

## 4. The LFA-1–Kindlin-3–IL-2Rβ Axis in Cytokine Responsiveness

IL-2 and interleukin-15 (IL-15) share the signalling receptor subunits IL-2Rβ (CD122) and the common γ chain (γc; CD132), but use distinct cytokine-specific α chains, interleukin-2 receptor α (IL-2Rα; CD25) and interleukin-15 receptor α (IL-15Rα), respectively [66,67]. This shared receptor architecture enables both cytokines to engage overlapping Janus kinases 1 and 3 (JAK1 and JAK3), signal transducer and activator of transcription 5 (STAT5), phosphoinositide 3-kinase–AKT (PI3K–AKT) and mitogen-activated protein kinase (MAPK) pathways, although their biological effects differ because of receptor distribution and modes of cytokine presentation [67]. IL-15 is particularly distinct in being commonly delivered in trans from IL-15Rα-bearing cells to neighbouring cells expressing IL-2Rβ and γc [68].

Spatial organisation of IL-2 receptor signalling has also been observed during homotypic T-cell interactions. At integrin LFA-1/ICAM-1-dependent contact sites, IL-2 secretion is polarised, and IL-2 receptor components accumulate towards the synaptic region, promoting efficient IL-2 capture and STAT5 activation [69]. Kindlin-3 has separately been shown to contribute to integrin LFA-1-dependent immune synapse formation between T cells and antigen-presenting cells [70]. Whether kindlin-3 also contributes to IL-2 receptor recruitment at homotypic T-cell synapses remains unknown.

Recent work has provided a potential molecular link between kindlin-3 and IL-2Rβ. In a HEK293 reconstitution system, the F0 subdomain of kindlin-3 binds residues 530–551 of the IL-2Rβ cytoplasmic domain [13]. Together with its F3 subdomain that interacts with the β2 subunit of the integrin LFA-1 [52,53], kindlin-3 promotes LFA-1–IL-2Rβ colocalisation at sites of integrin LFA-1 engagement in MOLT-4 acute lymphoblastic leukaemia cells. In addition, kindlin-3 regulates IL-2 signalling by promoting the persistence of IL-2Rβ at the cell surface, while depletion of kindlin-3 in NK92 natural killer (NK) cells reduces IL-2Rβ surface retention and accelerates receptor internalisation following IL-2 stimulation [13].

**Figure 3:**
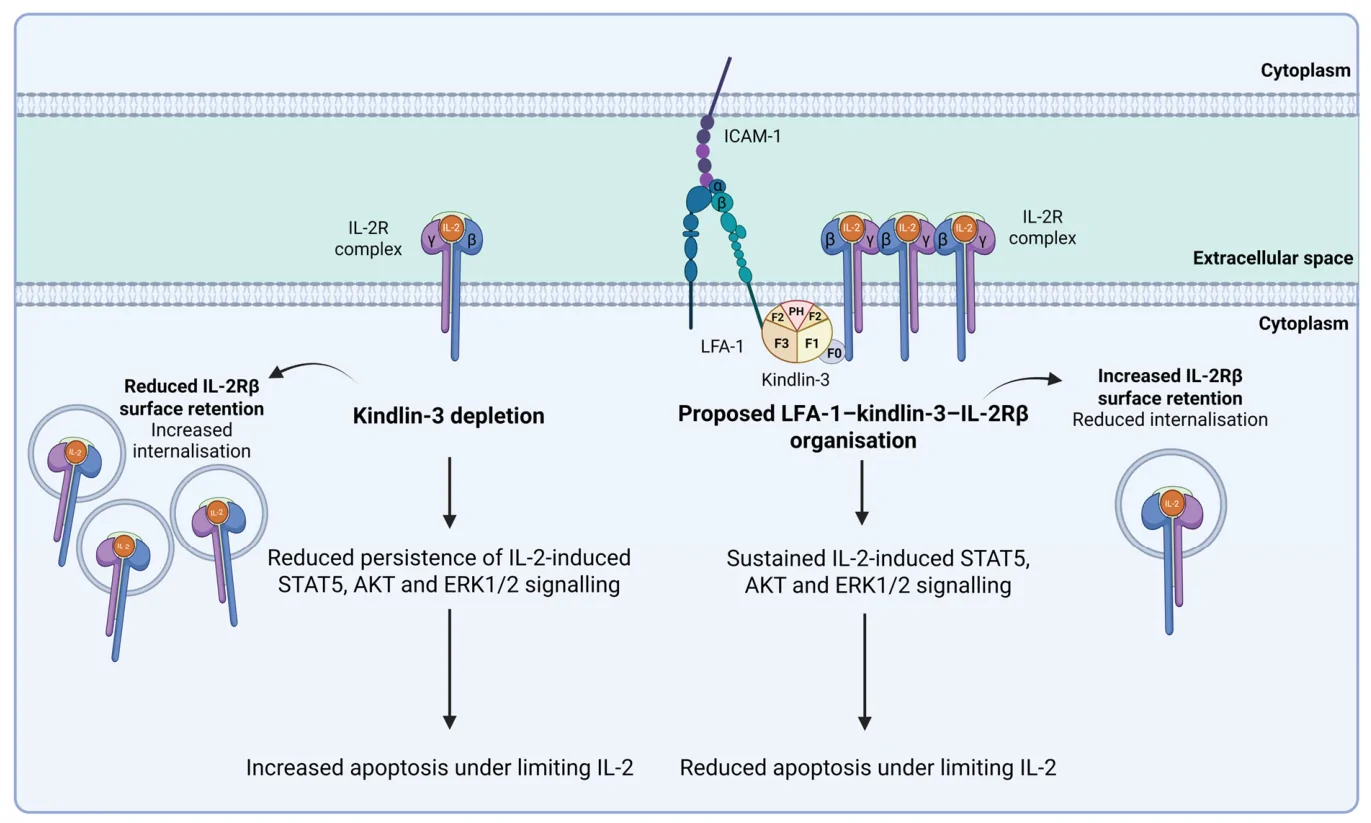
Proposed model for kindlin-3-mediated regulation of IL-2Rβ surface retention and sustained IL-2 signalling. Kindlin-3 interacts with the β2 integrin cytoplasmic tail through its F3 subdomain and with IL-2Rβ through its F0 subdomain, providing a molecular basis for the proposed spatial organisation of integrin LFA-1 and IL-2Rβ at the plasma membrane. In the presence of kindlin-3, integrin LFA-1-dependent retention of IL-2Rβ is increased and receptor internalisation is reduced, supporting sustained IL-2-induced STAT5, AKT and ERK1/2 signalling and reduced apoptosis under limiting IL-2 conditions (right panel). Conversely, kindlin-3 depletion reduces IL-2Rβ surface retention, increases receptor internalisation and accelerates the loss of IL-2-induced STAT5, AKT and ERK1/2 signalling, with a corresponding increase in apoptosis under limiting IL-2 conditions (left panel). Abbreviations: IL-2Rβ, interleukin-2 receptor β; IL-2, interleukin-2; LFA-1, lymphocyte function-associated antigen 1; STAT5, signal transducer and activator of transcription 5; ERK1/2, extracellular signal-regulated kinase 1/2. Created in BioRender. Teng, Z.-K. (2026) https://BioRender.com/nb4d2ne (accessed on 6 September 2026).

The effects of kindlin-3 on receptor retention are accompanied by changes in the persistence of IL-2 signalling. Following transient IL-2 exposure and subsequent cytokine withdrawal, depletion of kindlin-3 causes STAT5, AKT and extracellular signal-regulated kinase 1/2 (ERK1/2) phosphorylation to decay more rapidly in NK92 cells [13]. Given that STAT5 is a major downstream effector of IL-2Rβ/γc signalling that controls transcriptional programmes involved in lymphocyte proliferation and survival [67,71], reduced receptor residence could limit the duration of cytokine responsiveness. Consistent with this interpretation, kindlin-3-depleted NK92 cells exhibit increased apoptosis under limiting IL-2 conditions [13]. These findings support a role for kindlin-3 in sustaining IL-2 signalling by promoting IL-2Rβ surface persistence, as summarised in Figure 3.

Since IL-2Rβ is also an essential component of the IL-15 receptor, this model raises the testable possibility that kindlin-3 may influence IL-15 responsiveness, particularly in NK cells and memory CD8+ T cells that depend strongly on IL-15 for survival and homeostasis [67]. However, this has not been established, and the distinct biology of IL-15 trans-presentation may impose different requirements for receptor organisation [67,68]. Testing IL-15 responsiveness will therefore determine whether this axis is specific to IL-2 or represents a broader mechanism coupling integrin organisation to cytokine responsiveness.

## 5. Structural Determinants of LFA-1–Kindlin-3–IL-2Rβ Organisation

The proposed role of kindlin-3 in coordinating integrin LFA-1 with IL-2Rβ is likely to depend not only on its interactions with individual binding partners but also on its orientation and residence at the plasma membrane. The modular architecture of kindlin-3 provides several potential mechanisms. As summarised in Figure 1, the F0, F1, PH and F3 regions provide distinct protein- and membrane-interaction surfaces relevant to receptor organisation. In addition to the F0 subdomain, which can interact directly with IL-2Rβ as discussed above, the F3 subdomain binds integrin β cytoplasmic tails, while the F1 loop and PH domain provide additional interactions with membrane lipids [38,41,42,52,53,61,72]. Together, these features could orient kindlin-3 at the inner leaflet of the plasma membrane and stabilise its association with receptor-containing membrane domains.

The principal integrin-binding interface of kindlin-3 resides within its F3 subdomain. Biophysical studies have shown that kindlin-3 recognises the membrane-distal region of integrin β cytoplasmic tails containing the NPxY/F motif [53]. However, residues within the adjacent Ser/Thr-rich region preceding the membrane-distal motif, particularly in the β1A and β1D cytoplasmic tails, also contribute to kindlin recognition [53,73]. Structural studies of kindlin–integrin complexes subsequently defined the molecular interface through which the F3 subdomain engages this membrane-distal region [52]. The importance of binding-site accessibility is further demonstrated by the full-length kindlin-3 structure, in which the F3 integrin-binding pocket is masked by the PH domain of an adjacent protomer in the homotrimeric state, thereby preventing integrin-tail engagement [72]. In the context of the proposed relationship between integrin LFA-1, kindlin-3 and IL-2Rβ, F3-mediated binding to the β2 integrin cytoplasmic tail could therefore provide an integrin-specific anchor for kindlin-3. Since the F0 subdomain provides a distinct interaction surface for IL-2Rβ, these spatially separated binding sites could permit kindlin-3 to engage the two receptors within the same membrane region. Such an arrangement could contribute to the integrin LFA-1-dependent retention of IL-2Rβ described above, although simultaneous engagement of both receptors by kindlin-3 in intact cells has not yet been demonstrated.

The F1 loop may provide an additional membrane anchor. All three kindlins contain an extended insertion within F1 that includes a conserved polylysine-rich sequence. Mutation or deletion of this motif in kindlin-1 and kindlin-2 compromises membrane association and integrin-dependent activation, supporting a conserved role for the F1 loop in membrane interaction [42]. Nuclear magnetic resonance (NMR) analysis of the corresponding 83-residue loop of kindlin-3 subsequently demonstrated preferential interaction with vesicles containing negatively charged phospholipids, with its positively charged N-terminal region contributing to membrane association [41]. These interactions could provide an additional electrostatic contact between kindlin-3 and the inner leaflet of the plasma membrane. Although a role for the F1 loop in IL-2Rβ regulation has not been demonstrated directly, increased membrane residence could favour repeated or prolonged encounters between membrane-associated kindlin-3, integrin LFA-1 and IL-2Rβ, thereby increasing the probability of their local association.

A second lipid-sensitive route to membrane recruitment is provided by the PH domain. The kindlin-3 PH domain preferentially interacts with phosphatidylinositol-(3,4,5)-trisphosphate (PIP3) over phosphatidylinositol-(4,5)-bisphosphate (PIP2), and deletion of the PH domain markedly reduces kindlin-3 localisation at the B-cell plasma membrane and prevents integrin LFA-1-dependent adhesion and migration [61]. Structural and biophysical analyses subsequently confirmed direct lipid binding by the kindlin-3 PH domain, including high-affinity association with PIP3-containing membranes [38]. More recent super-resolution imaging has also implicated the PH domain in kindlin-3-dependent integrin LFA-1 and Mac-1 clustering following IL-8 or fMLP stimulation [60]. The PH domain may therefore do more than increase general membrane affinity. By favouring recruitment to phosphoinositide-enriched regions, it could position kindlin-3 within membrane domains that are permissive for receptor clustering and signalling.

Together, these domains suggest a multivalent mechanism through which kindlin-3 could contribute to receptor organisation at the plasma membrane. F3-mediated integrin binding and F0-mediated IL-2Rβ binding could provide receptor-specific contacts, while the F1 loop and PH domain could increase membrane residence and spatial confinement. Whether these membrane-binding regions directly regulate the formation, organisation or persistence of a proposed integrin LFA-1–kindlin-3–IL-2Rβ assembly remains unknown. Selective mutational analysis combined with measurements of receptor colocalisation, surface retention and sustained cytokine signalling would provide a direct test of this model.

## 6. Post-Translational Regulation of Kindlin-3: Phosphorylation as a Potential Regulator of Receptor Organisation

Phosphorylation provides an additional layer of kindlin-3 regulation. The reported Thr482/Ser484 phosphorylation sites are indicated in Figure 1. Stimulation of human platelets and human erythroleukaemia (HEL) megakaryocytic-like cells induces phosphorylation at Thr482 and/or Ser484, residues within a region not conserved in kindlin-1 or kindlin-2 [74]. Subsequent analysis using a phospho-specific antibody identified Ser484 as a major phosphorylation site whose modification increases rapidly and transiently following platelet activation [75]. Mutation of Thr482 and Ser484 impaired integrin αIIbβ3-dependent responses in HEL cells [74], while expression of the T482A/S484A mutant in mouse megakaryocytes and MDA-MB-231 breast cancer cells impaired integrin activation and other cellular responses [75]. The physiological importance of this modification was subsequently demonstrated in mice carrying the corresponding non-phosphorylatable S485A substitution, which showed impaired platelet integrin αIIbβ3 activation, aggregation and adhesion, together with prolonged bleeding and delayed arterial thrombosis [76].

Phosphorylation of kindlin-3 also contributes to β2 integrin regulation in leukocytes. In neutrophils and neutrophil-like HL-60 cells, integrin-linked kinase (ILK) promotes membrane recruitment and activation of protein kinase C (PKC), while chemokine stimulation induces ILK-dependent phosphorylation of kindlin-3. Genetic or experimental disruption of this pathway impairs formation of the high-affinity conformation of integrin and reduces neutrophil adhesion and recruitment [77]. These findings support phosphorylation as a stimulus-responsive mechanism for modulating kindlin-3 function during β2 integrin regulation. The molecular consequences of this modification remain incompletely defined. Structural studies captured kindlin-3 in an autoinhibited homotrimeric state in which the F3 integrin-binding site is occluded, and phosphorylation has been proposed as one possible mechanism regulating trimer formation or dissociation, although this has not been experimentally established [72].

Whether phosphorylation also regulates the proposed relationship between integrin LFA-1, kindlin-3 and IL-2Rβ remains unknown. By altering kindlin-3 conformation, membrane localisation or interaction dynamics, phosphorylation could influence its recruitment to or residence within integrin LFA-1-associated membrane regions and thereby affect IL-2Rβ retention. Whether phosphorylation changes kindlin-3 binding to IL-2Rβ, the lifetime or stoichiometry of the proposed receptor assembly, or the persistence of cytokine signalling remains unknown [13]. These questions provide a direct route for testing whether post-translational modification acts as a regulatory switch for receptor organisation.

## 7. Outstanding Questions and Perspectives

Several mechanistic questions remain regarding the proposed relationship between integrin LFA-1, kindlin-3 and IL-2Rβ. Most importantly, it remains unknown whether these proteins form a stable ternary assembly in intact cells, what its stoichiometry and lifetime might be and whether their association is constitutive or dynamically regulated. The F1 loop, PH domain and phosphorylation of kindlin-3 provide potential mechanisms for regulating membrane recruitment or receptor association, as discussed above, but their contributions to IL-2Rβ retention have not been established. Selective perturbation of these regions, combined with measurements of receptor proximity, trafficking and signalling kinetics, should help distinguish direct receptor-organising functions of kindlin-3 from broader consequences of altered integrin regulation.

The biological scope of this mechanism also remains unclear. IL-2Rβ is shared by the receptors for IL-2 and IL-15 [66,67], yet the functional consequences of kindlin-3-dependent IL-2Rβ retention have so far been characterised primarily in the context of IL-2 [13]. Determining whether kindlin-3 also regulates responses to physiologically trans-presented IL-15 will therefore be important, particularly in NK cells and CD8+ T cells. Comparative studies in primary NK cells, conventional and regulatory T cells, and other IL-2Rβ-expressing populations could establish whether this mechanism is broadly utilised or depends on cell type, activation state or receptor abundance. This question may also be relevant to lymphoid malignancies in which IL-2 or IL-15 signalling contributes to cellular survival or proliferation [78,79,80], although there is currently no direct evidence that kindlin-3-dependent IL-2Rβ retention contributes to cytokine responsiveness in leukaemia or lymphoma. Reported expression of individual components of the proposed LFA-1–kindlin-3–IL-2Rβ axis in selected lymphoid malignancies is summarised in Table 2.

An additional question is whether the regulation of non-integrin receptors is conserved across the kindlin family. The F0 domains of all three kindlins can interact with the cytoplasmic region of IL-2Rβ in vitro [13], but the functional significance of these interactions outside haematopoietic cells is unknown. Kindlin-1 provides one potential context for testing this possibility. Loss of kindlin-1 disrupts intestinal epithelial integrity and is associated with intestinal inflammation [96,97], while IL-2Rβ-containing receptor components and IL-15-responsive signalling have been reported in intestinal epithelial cells [98,99]. Whether kindlin-1 regulates IL-2Rβ trafficking or signalling in this setting remains entirely untested. Kindlin-2 provides a complementary precedent, as its established interactions with non-integrin receptors and its ability to stabilise receptor-containing membrane complexes indicate that receptor-organising functions are not restricted to kindlin-3 [6,23]. Comparative studies across the three paralogues may therefore determine whether receptor regulation represents a broader property of the kindlin family or a collection of tissue- and receptor-specific functions.

Kindlin-2 is endogenously expressed in the nervous system, and its depletion impairs integrin signalling and axon extension in sensory neurons [100]. This raises the question of whether kindlin-2 may also participate in the broader crosstalk between β1 integrins and growth factor or developmental receptor pathways in neural cells. In neural progenitors, β1 integrin, Notch1 and EGFR signalling are functionally coordinated [101], while in ependymal stem cells β1 integrin directly associates with bone morphogenetic protein receptor type 1A (BMPR1A) and type 1B (BMPR1B) and regulates bone morphogenetic protein (BMP) receptor localisation and downstream signalling after spinal cord injury [102]. Brain-derived neurotrophic factor (BDNF) signalling can likewise induce rapid β1 integrin clustering in neuronal growth cones, which is required for neurotrophin-stimulated axon extension [103]. Although it remains unknown whether kindlin-2 physically couples β1 integrins to these receptors in central nervous system (CNS) cells, its ability to participate in β1 integrin-containing receptor assemblies in other tissues raises the possibility that analogous mechanisms may contribute to the integration of extracellular matrix and growth factor signals in neural contexts [23].

**Figure 4:**
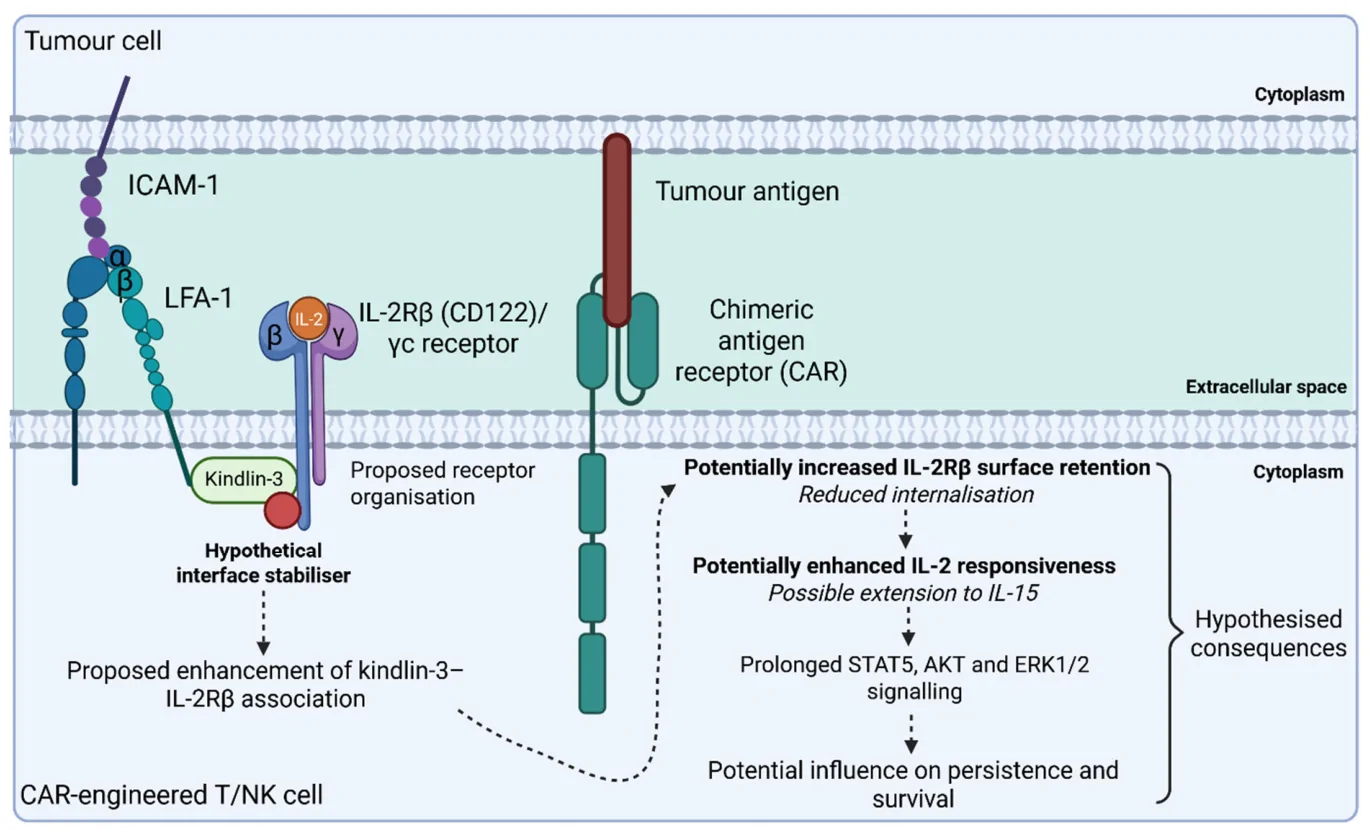
Conceptual hypothesis for modulating kindlin-3–IL-2Rβ association in engineered lymphocytes. This schematic illustrates a speculative model in which strengthening the kindlin-3–IL-2Rβ interaction could increase cytokine responsiveness in CAR-engineered T or NK cells. Alongside CAR-mediated tumour-antigen recognition and integrin LFA-1–ICAM-1-dependent adhesion, enhanced kindlin-3–IL-2Rβ association is proposed to increase IL-2Rβ surface retention and reduce receptor internalisation. If the kindlin-3-dependent retention mechanism also extends to IL-15 receptor signalling, this could in principle increase responsiveness to IL-2 and IL-15; prolong STAT5, AKT and ERK1/2 signalling; and influence lymphocyte persistence or survival. The red circle represents a hypothetical interface stabiliser of the kindlin-3–IL-2Rβ interface. Dashed arrows indicate proposed relationships that have not been experimentally demonstrated. No pharmacological stabiliser of this interaction has yet been established. Abbreviations: CAR, chimeric antigen receptor; NK, natural killer; LFA-1, lymphocyte function-associated antigen 1; ICAM-1, intercellular adhesion molecule 1; IL-2Rβ, interleukin-2 receptor β; IL-2, interleukin-2; IL-15, interleukin-15; STAT5, signal transducer and activator of transcription 5; ERK1/2, extracellular signal-regulated kinase 1/2. Created in BioRender. Teng, Z.-K. (2026) https://BioRender.com/c0zf2vx (accessed on 6 September 2026).

Defining the molecular interfaces responsible for these functions may have translational implications. Broad inhibition of kindlin-3 or integrin LFA-1 would interfere with essential integrin-dependent immune functions [10,12], limiting the potential for selective targeting. If the interaction between kindlin-3 and IL-2Rβ can be manipulated independently of canonical integrin regulation, selective modulation of cytokine-receptor retention is possible. Persistence and cytokine responsiveness are important determinants of chimeric antigen receptor (CAR)-T and CAR-NK function, and IL-15-based engineering strategies have been used to enhance these properties [104,105]. If kindlin-3-dependent IL-2Rβ retention is found to extend to IL-15 signalling, its modulation may influence cytokine sensitivity or persistence in these cells (Figure 4).

## 8. Methodology

This review was supported by a structured literature search of PubMed. The search was conducted between June and September 2026 using combinations of terms relating to kindlin family members, integrin regulation, receptor signalling, membrane trafficking, receptor stability, phosphorylation and cytokine signalling. The search strategy was:

(kindlin[Title/Abstract] OR kindlin-1[Title/Abstract] OR kindlin-2[Title/Abstract] OR kindlin-3[Title/Abstract] OR FERMT1[Title/Abstract] OR FERMT2[Title/Abstract] OR FERMT3[Title/Abstract]) AND (integrin*[Title/Abstract] OR receptor*[Title/Abstract] OR signal*[Title/Abstract] OR traffick*[Title/Abstract] OR stabil*[Title/Abstract] OR membrane*[Title/Abstract] OR phosphorylation[Title/Abstract] OR cytokine*[Title/Abstract] OR scaffold*[Title/Abstract]).

The search was restricted to English-language publications and preprints were excluded. Primary research articles were considered where they provided molecular, biochemical, cellular, animal or human evidence relevant to kindlin-mediated integrin regulation, receptor trafficking or stability, membrane organisation, signalling interactions, post-translational regulation or loss-of-function phenotypes. Review articles, editorials and other secondary literature were not included in the primary-study pool but were retained where useful for contextual information and citation searching. Studies in which kindlins were mentioned only incidentally, or which lacked substantive mechanistic or biological relevance to the scope of this review, were excluded.

**Figure 5:**
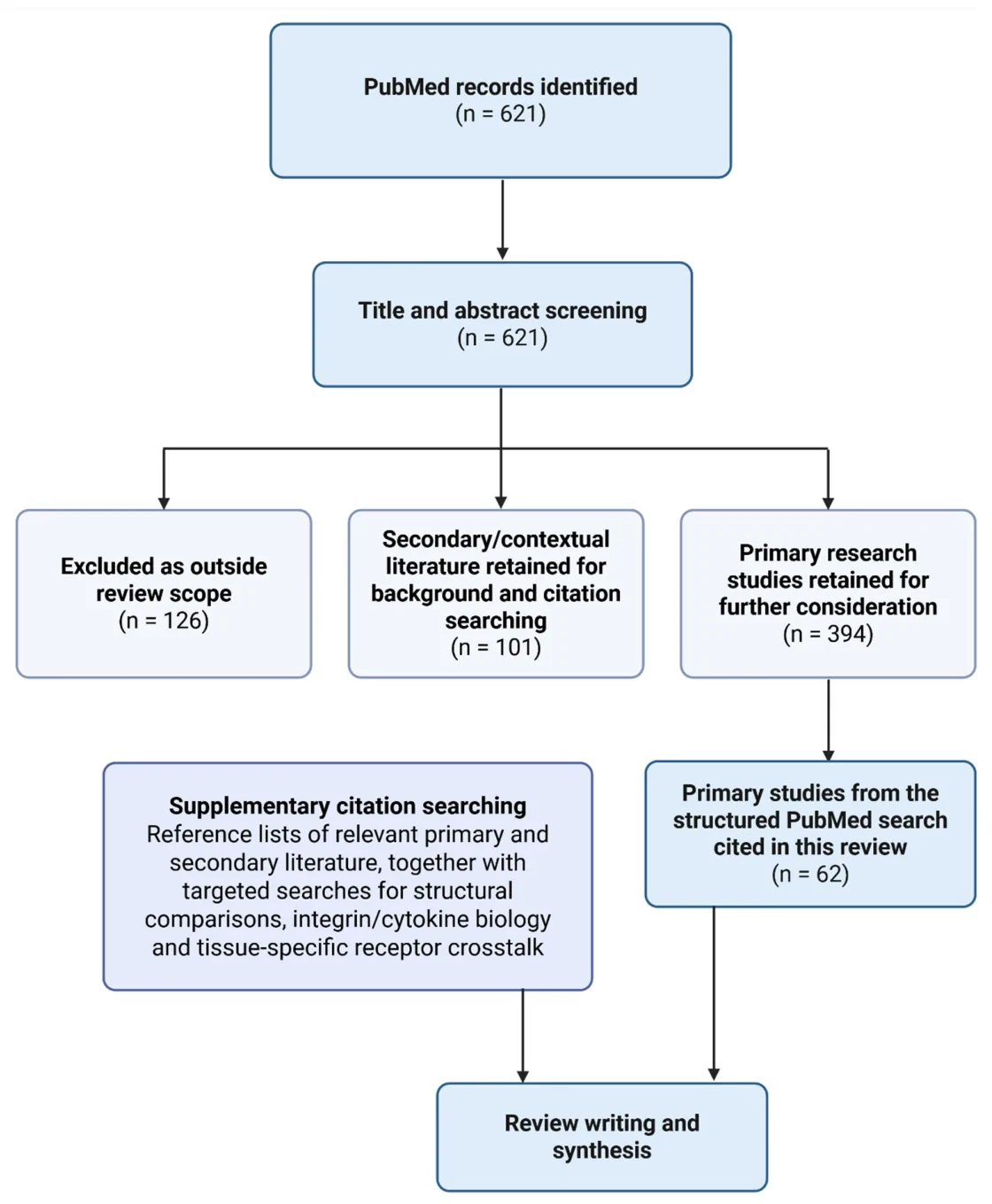
Literature identification and screening process supporting this review. A structured PubMed search identified 621 records. Following title and abstract screening, 126 records were excluded as outside the scope of this review, 101 secondary or contextual articles were retained for background information and citation searching, and 394 primary research studies were retained for further consideration. Of these, 62 primary studies from the structured PubMed search were cited in this review. Supplementary citation searching of relevant primary and secondary literature, together with targeted searches for structural comparisons, integrin and cytokine biology and tissue-specific receptor crosstalk, provided additional references incorporated into this review.

The search identified 621 records. Following title and abstract screening, 126 records were excluded as outside the scope of this review, 101 secondary or contextual articles were retained for background information and citation searching and 394 primary studies were retained for further consideration. These studies were subsequently prioritised according to their relevance to the major themes of this review, including integrin regulation, receptor organisation and trafficking, signalling interactions and kindlin regulation. Of the 394 retained primary studies, 62 are cited in the present review. Additional references were identified through supplementary citation searching to provide contextual evidence on integrin biology, cytokine signalling, structural comparisons with talin and ERM proteins, and tissue-specific receptor crosstalk. The literature identification and screening process is summarised in Figure 5.

## 9. Conclusions

Kindlin-3 has well-established roles in haematopoietic integrin regulation, contributing to integrin activation, clustering and integrin-associated signalling. More recent evidence suggests that these functions may extend to cytokine receptor organisation, with kindlin-3 promoting integrin LFA-1-dependent retention of IL-2Rβ at the plasma membrane and sustaining IL-2 signalling. Whether kindlin-3 similarly regulates IL-15 signalling remains unknown, despite the shared use of IL-2Rβ by the IL-2 and IL-15 receptors. Likewise, potential roles in cytokine-dependent lymphoid malignancies and in modulating the persistence or cytokine responsiveness of CAR-T and CAR-NK cells remain to be tested. Collectively, these observations support a broader framework in which kindlin-3 functions not only in integrin regulation but may also contribute to the context-dependent organisation of receptor signalling at the plasma membrane.

## Figures and Tables

**Table 1:** Reported protein partners of kindlin family members.

Kindlin	Non-Integrin Partner	Reported Functional Consequence	Relationship to Integrin Function	Experimental Context/Model	References
1	Epidermal growth factor receptor (EGFR)	Stabilises EGFR by limiting lysosomal degradation and supports EGF-dependent signalling and migration.	Non-integrin-associated *	Kindler syndrome patient skin and primary keratinocytes	[7]
	Polo-like kinase 1 (PLK1)	Regulates centrosomal localisation and mitotic spindle assembly through interaction with PLK1.	Integrin-associated	MDA-MB-231 cells; MCF-10A and HeLa cells; mouse keratinocytes	[14]
	Transforming growth factor β receptor I (TβRI), Smad anchor for receptor activation (SARA), Smad3	Promotes assembly of a TβRI-SARA-Smad3 signalling complex and TGF-β/Smad3 signalling.	Non-integrin-associated *	HCT116 and SW1116 colorectal cancer cells; mouse xenografts; human colorectal cancer tissues	[15]
2	Paxillin	Recruits paxillin and Arp2/3 to nascent adhesions, promoting protrusion and cell spreading.	Integrin-associated	Mouse kidney fibroblasts	[16]
	Migfilin	Regulates migfilin recruitment and dynamics at focal adhesions.	Integrin-associated	NIH3T3 fibroblasts and primary keratinocytes	[17]
	Integrin-linked kinase (ILK)	Promotes focal-adhesion localisation and cell spreading through binding to the ILK pseudokinase domain.	Integrin-associated	HeLa, OVCAR-3 and Chinese hamster ovary (CHO) integrin αIIbβ3 cells	[18]
	Actin	Directly binds F-actin and supports cytoskeletal organisation and integrin outside-in signalling.	Integrin-associated	Kindlin-2+/− mouse endothelial cells; C2C12 and CHO integrin αIIbβ3 cells	[19]
	Src	Promotes Src-dependent paxillin phosphorylation, migration and proliferation following matrix adhesion.	Integrin-associated	Mouse embryonic fibroblasts and human podocytes	[20]
	Girdin	Enhances β1 integrin recognition, activation and clustering, promoting adhesion and invasion.	Integrin-associated	MDA-MB-231, HeLa and COS-7 cells	[21]
	Epidermal growth factor receptor (EGFR)	Stabilises EGFR by limiting ubiquitination and degradation, supporting EGF-dependent migration.	Non-integrin-associated *	SUM159 and MDA-MB-231 breast cancer cells	[6]
	Transforming growth factor β receptor I (TβRI)	Promotes TβRI-Smad3 signalling and, in triple-negative breast cancer (TNBC), stabilises a β1 integrin–kindlin-2–TβRI receptor complex.	Context-dependent	Human renal tubular epithelial cells and renal fibrosis models; MDA-MB-231 and 4T1 TNBC cells and mouse tumour models	[22,23]
	β-catenin and T-cell factor 4 (TCF4)	Forms a transcriptional complex that enhances Wnt-responsive gene expression.	Non-integrin-associated *	Breast cancer cells; HEK293T and renal epithelial cells	[24]
	Y-box-binding protein 1 (YB-1) and β-catenin	Enhances EGFR transcription through a kindlin-2/YB-1/β-catenin complex.	Non-integrin-associated *	U-87 MG glioma cells; mouse tumour models; human glioma tissues	[25]
	Androgen receptor (AR) and Src	Scaffolds AR and Src to promote AR phosphorylation and signalling.	Non-integrin-associated *	BT549 and MDA-MB-453 breast cancer cells; mouse mammary tumour models	[26]
	MOB kinase activator 1 (MOB1) and praja2	Promotes praja2-mediated MOB1 degradation and enhances YAP signalling.	Non-integrin-associated *	Human renal epithelial and kidney-derived cell lines; unilateral ureteral obstruction (UUO) mouse model; human renal fibrosis specimens	[27]
	Rho GDP-dissociation inhibitor α (RhoGDIα)	Stabilises the RhoGDIα-Rac1 complex and restrains Rac1 activation in podocytes.	Non-integrin-associated *	Podocyte-specific kindlin-2-deficient mice and cultured human podocytes	[28]
	Four-and-a-half LIM domain protein 1 (FHL1)	Recruits FHL1 to focal adhesions and limits Src-dependent FHL1 phosphorylation and nuclear translocation.	Integrin-associated	HeLa cells; mouse embryonic fibroblasts; human lung adenocarcinoma tissues	[29]
	DNA methyltransferase 1 (DNMT1)	Stabilises DNMT1, promoting E-cadherin promoter methylation and transcriptional repression.	Non-integrin-associated *	Breast cancer cells and patient tissues; mammary-specific kindlin-2 transgenic mice	[30]
	Smad ubiquitination regulatory factor 1 (Smurf1)	Promotes kindlin-2 ubiquitination and degradation, thereby limiting integrin activation.	Integrin-associated	CHO integrin αIIbβ3 cells; NIH3T3 and mouse embryonic fibroblasts; human colon cancer tissues	[31]
	Clathrin heavy chain	Regulates endothelial CD39/CD73 endocytosis and recycling, influencing haemostasis.	Non-integrin-associated *	Kindlin-2+/− mouse endothelial cells; human umbilical vein endothelial cells (HUVECs); kindlin-2+/− mice	[8]
3	Receptor for activated C kinase 1 (RACK1)	Couples integrin LFA-1 engagement to membrane-proximal signalling.	Integrin-associated	SKW3 T-lymphoma cells; K562 transfectants; human T-cell lymphoblasts.	[32]
	Adhesion and Degranulation-promoting Adapter Protein (ADAP)	Promotes integrin αIIbβ3 activation and stable fibrinogen binding through kindlin-3/talin-associated complexes.	Integrin-associated	Human and ADAP-deficient mouse platelets; CHO integrin αIIbβ3 reconstitution model	[33]
	Leupaxin	Recruits leupaxin to podosomes and promotes podosome stability through paxillin dephosphorylation.	Integrin-associated	Kindlin-3 hypomorphic and knockout mouse preosteoclasts/osteoclasts	[34]
	Paxillin	Supports integrin αIIbβ3 signalling through direct interaction with kindlin-3.	Integrin-associated	Human and mouse platelets; CHO integrin αIIbβ3 cells	[35]
	Interleukin-2 receptor β (IL-2Rβ)	Promotes integrin LFA-1-dependent IL-2Rβ surface retention and sustains IL-2 signalling.	Integrin-associated	MOLT-4 acute lymphoblastic leukaemia cells; NK92 natural killer cells; HEK293 reconstitution system	[13]

“Context-dependent” denotes interactions for which integrin involvement differs between experimental settings. * “Non-integrin-associated” indicates that the reported function has not been linked to integrin regulation in the cited study.

**Table 2:** Reported expression of kindlin-3, integrin LFA-1 and IL-2Rβ in selected lymphoid malignancies.

Malignancy	Material Examined	Kindlin-3	LFA-1	IL-2Rβ	References
Adult T-cell leukaemia/lymphoma (ATL/ATLL)	Primary patient cells	Not specifically examined	Reported	Reported; heterogeneous between studies	[81,82,83]
	ATL-derived cell lines	Not specifically examined	Reported; expression varies between cell lines	Functional IL-2Rβ signalling demonstrated in IL-2/IL-15-responsive cell lines	[84,85]
Cutaneous T-cell lymphoma (CTCL), including Sézary syndrome	Primary patient cells	Not specifically examined	Reported; markedly reduced in some Sézary cell populations	*IL2RB* transcript detected in a subset of patient samples	[86,87]
	CTCL-derived cell lines	Not specifically examined	Not specifically examined	Functional IL-2Rβ signalling demonstrated in cytokine-responsive cell lines	[88]
T-cell large granular lymphocytic leukaemia (T-LGLL)	Primary patient cells	Not specifically examined	Not specifically examined	Reported; circulating malignant cells express CD122	[89]
	T-LGLL-derived cell lines	Not specifically examined	Not specifically examined	Functional IL-2Rβ signalling demonstrated in MOTN-1 cells	[90]
Chronic lymphocytic leukaemia (CLL)	Primary patient cells	Reported as MIG2B (*FERMT3*) by plasma-membrane proteomics	Reported; generally reduced compared with normal lymphocytes	Reported in a subset; expression is heterogeneous	[83,91,92]
	CLL-derived cell lines	Not specifically examined	Reported in MEC1 and MEC2 cells (CD11a+CD18+)	Not specifically examined	[93]
Hairy cell leukaemia (HCL)	Primary patient cells	Not specifically examined	Reported at low or undetectable levels in many cases	Reported in classic and variant HCL	[94,95]
	HCL-derived cell lines	No suitable evidence identified	No suitable evidence identified	No suitable evidence identified	

Expression data were derived from primary patient material or established malignancy-derived cell lines, as indicated. “Not specifically examined” indicates that direct disease- or model-specific evidence was not identified in the literature reviewed and should not be interpreted as absence of expression. Where direct protein-expression data were unavailable, transcript-level or functional evidence is indicated explicitly. Studies reporting individual components were generally performed independently; therefore, the table does not establish their simultaneous expression or functional coupling within the same malignant cells.

## Data Availability

No new data were created or analysed in this study. Data sharing is not applicable to this article.
